# 3D plasmonic crystal metamaterials for ultra-sensitive biosensing

**DOI:** 10.1038/srep25380

**Published:** 2016-05-06

**Authors:** Andrey I. Aristov, Maria Manousidaki, Artem Danilov, Konstantina Terzaki, Costas Fotakis, Maria Farsari, Andrei V. Kabashin

**Affiliations:** 1 Aix Marseille University, CNRS, LP3 UMR 7341, Marseille cedex 9, Campus de Luminy - Case 917, 13288, France; 2 IESL-FORTH, N. Plastira 100, Heraklion, 70013, Crete, Greece; 3 Department of Material Science and Technology, University of Crete, Heraklion, Greece; 4 Department of Physics, University of Crete, Heraklion, Greece

## Abstract

We explore the excitation of plasmons in 3D plasmon crystal metamaterials and report the observation of a delocalized plasmon mode, which provides extremely high spectral sensitivity (>2600 nm per refractive index unit (RIU) change), outperforming all plasmonic counterparts excited in 2D nanoscale geometries, as well as a prominent phase-sensitive response (>3*10^4^ deg. of phase per RIU). Combined with a large surface for bioimmobilization provided by the 3D matrix, the proposed sensor architecture promises a new important landmark in the advancement of plasmonic biosensing technology.

Having a variety of critically important applications in biomedical research, plasmonic biosensors form the core of current label-free biosensing technology to study biomolecular binding events between a target analyte from an aqueous solution and its corresponding receptor immobilized on a solid/liquid interface^1^,^2^. Plasmonic transduction relies on the refractive index (RI) control of the binding and profits from the difference of refractive indices between biological molecules and the aqueous environment (n_bio_ =  1.45–1.55 Refractive Index Units (RIU) compared to n_wat_ =  1.33 RIU). Confined to a metal/liquid interface, surface plasmons are critically dependent on the RI of the liquid medium within the penetration depth of the evanescent field and can be used to follow biomolecular interactions on the metal surface. In this case, one can avoid expensive, time-consuming and precision-interfering labelling steps to mark analytes and control binding/recognition events in real-time^2^,^3^,^4^, while the high sensitivity of this transduction is due to the strong plasmon-mediated electric field probing the molecules^5^.

Conventional plasmonic biosensors employ Kretschmann-Raether prism geometry to excite surface plasmon polaritons over a thin 50-nm gold film in conditions of Surface Plasmon Resonance (SPR)^3^,^4^. Such SPR biosensors can provide relatively high sensitivity of the order of 2000–4000 nm of spectral resonance shift per refractive index unit (RIU) change^4^, but this geometry cannot satisfy many modern trends of biosensing advancing towards new biochemical nano-architectures, resolution beyond the diffraction limit, size-based selectivity etc^6^. In contrast, plasmons excited over nanostructures, including localized plasmon resonances (LPR) over 2D periodic nanoparticle arrays^7^,^8^, diffraction-coupled LPR (DC-LPR) over 2D nanoparticle arrays^9^,^10^,^11^,^12^,^13^, or surface plasmon polaritons over 2D nanohole arrays (NHA)^14^,^15^ can offer a series of promising biosensing nanoarchitectures and novel attractive functionalities. However, spectral sensitivity of 2D nanoscale geometries to refractive index variations is much lower: 200–500 nm/RIU for LPR and DC-LPR using nanoparticle arrays^7^,^9^,^11^,^13^, 300–400 nm/RIU for nanohole arrays^14^,^15^. The sensitivity problem of 2D periodic plasmonic arrays is related to diffractive nature of coupling light to plasmons, which links the sensitivity to structure periodicity Δ *λ/*Δ *n* ~ *d*^16^, which is typically of the order of hundreds of nm. The above-stated spectral sensitivities condition a detection limit of 1 pg/mm^2^ of surface coverage by biomaterial for SPR^3^,^4^ and of about 1 ng/mm^2^ for LPR^7^,^8^ and NHA^17^, which makes possible label-free studies of many interactions, but still inferior to labeling methods by more than 2 orders of magnitude.

The sensitivity of plasmonic transduction can be improved by employing the phase characteristics of light instead of the amplitude ones, to follow biomolecular interactions^18^,^5^,^19^, although such result can be achieved for a relatively narrow dynamic range of measurements^19^. Phase-sensitive schemes profit from a sharp phase jump, which always occurs with a sudden drop of light intensity (light darkness) at the point of the resonance^20^,^21^. The sharpness of this jump and corresponding phase sensitivity do not follow the periodicity-related limitation rule, but are determined by the quality of resonances and first of all their depth (light darkness in the resonance)^11^,^21^. As an example, due to a much lower light intensity under DC-LPR compared to SPR in the Kretschmann-Raether prism arrangement, phase sensitivity of DC-LPR can much exceed the relevant parameter for SPR promising potential improvement of plasmonic biosensing technology down to single molecule detection level^21^. The combination of ultrahigh point sensitivity (provided e.g., by the phase interrogation) and fairly high sensitivity for a wider dynamic range of measurements (provided by the spectral or angular interrogation) looks like a very attractive basis for the development of next generation plasmonic biosensors. However, this combination is hardly possible with current 2D nanoperiodic structures due to the periodicity limitation rule for the spectral sensitivity.

Here, we explore 3D geometries of plasmon excitation by employing designed plasmon crystal metamaterials. We show that such a transition to 3D nanoarchitectures leads to the excitation of a novel plasmon mode, resulting in very high sensitivities in both spectral (>2600 nm/RIU) and phase (>3*10^4^ deg. of phase per RIU) interrogations. On the other hand, such structures can offer a high surface-to-volume ratio for bioimmobilization, in order to increase the resulting sensitivity, as well as enable novel promising sensing functionalities.

## Results and Discussion

**Figure 1:**
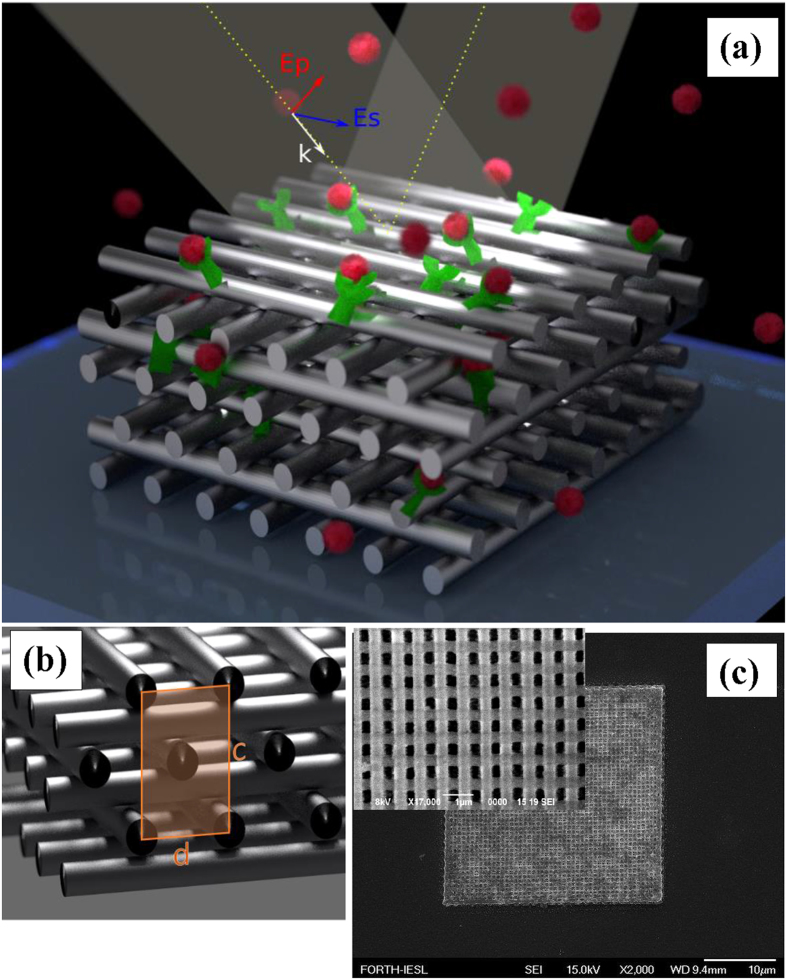
3D plasmonic metamaterial based on Ag-coated woodpile crystal. (**a**) Schematic illustration of biosensing using the metamaterial. A receptor (green) is immobilized inside the metalized woodpile structure, while its selective partner (red) binds into receptor sites, leading to a change of parameters for the reflected light; (**b**) Schematic representation of a Woodpile crystal. Unit cell is highlighted by orange; (**c**) Scanning electron microscopy of woodpile metamaterial structures produced by multiphoton laser polymerization, followed by Ag-based metallization.

One such possible design of a 3D plasmonic metamaterial is based on a classical woodpile photonic crystal geometry^22^, consisting of layers of one-dimensional rods with a stacking sequence that repeats itself every four layers, as shown in Fig. 1(a,b). The distance between four adjacent layers is *c* and, within each layer, the axes of the rods are parallel to each other with a distance *d* between them (Fig. 1b). The adjacent layers are rotated by 90°. Between every other layer, the rods are shifted relative to each other by *d*/2. For the case of 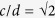, the lattice can be derived from a face-centered-cubic (fcc) unit cell with a basis of two rods. The woodpile structures were fabricated by direct laser writing (DLW) using multi-photon polymerization^23^ (see details in Methods section), followed by Ag-based electroless plating^24^. Typical scanning electron microscopy (SEM) images of 3D woodpile structures are shown in Fig. 1(c). The period of the structures and the diameter of metalized nanorods were 700 nm and 350 nm, respectively.

**Figure 2:**
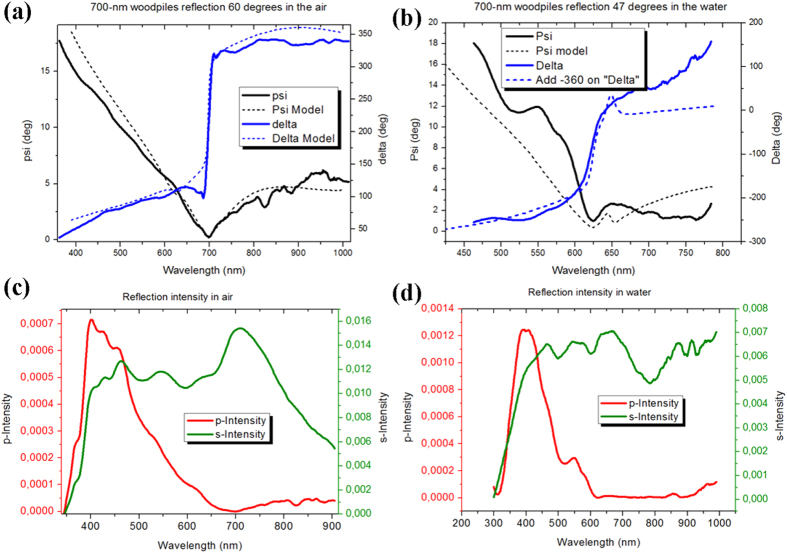
Phase singularities in 3D woodpile plasmonic metamaterials. Theoretical (dashed curved) and experimental (solid) spectral dependencies of ellipsometric reflection Ψ and phase Δ when the metamaterial structure is in contact with air (**a**) and water (**b**) environments (the data are given for angles of incidence that show typical spectral dependencies of Ψ and Δ for an angle of incidence of 60 deg. and 47 deg., respectively). The ellipsometric reflection becomes close to zero (topological darkness) at 700 nm and 625 nm, respectively, resulting in sharp jumps of phase Δ . Spectral dependences for p-polarized *R*_*p*_ (red) and s-polarized *R*_*s*_ (green) reflected intensities at the contact of metamaterial structure with air (**c**) and water (**d**) environments.

Our experiments showed that woodpile-based structure does not transmit light evidencing a complete domination of plasmonic phenomena over interference effects in photon crystals^25^. The structure still can demonstrate diffraction effects, but all diffraction beams are of very small intensity, several orders of magnitude smaller than the intensity of reflected light. In our tests, we examined properties of light reflected from the structured in zero diffraction order by using ellipsometry (Woollam M-2000 system). Ellipsometry enables the measurement not only of the amplitude parameter Ψ , which characterizes the ratio of amplitude reflection coefficients for light polarization parallel (*r*_*p*_) and perpendicular (*r*_*s*_) to the plane of incidence, but also the phase difference between the p- and s- components (Δ). As shown in Fig. 2(a,b), the light reflected from the woodpile metamaterial leads to the generation of deep resonances, obviously related to the excitation of plasmon modes over the metamaterial matrix. The resonances are observed at 700 nm and 625 nm when the structure is in contact with air or water environments, respectively, and the relevant angles of light incidence are 60 deg. and 56 deg. Figure 2(c,d) demonstrate the dependence for the reflectivity of p-polarized (*r*_*p*_) and s-polarized (*r*_*s*_) components of the light reflected from the metamaterial. One can see that the main resonant feature is generated by p-polarized light. Here, the resonances are characterized by a substantial absorption of light in the near-infrared region. It is important that for both air and water environments the light intensity in the resonances is very low. Indeed, the intensity within the *r*_*p*_ minimum does not exceed 10^−6^% and 10^−4^% for air and aqueous environments, respectively. As follows from the topological properties of phase^20^,^21^, such “light darkness” at the resonances should lead to singularities of phase of reflected light. Indeed, we could observe very sharp phase jumps when the metamaterial was in contact with air (Fig. 2a). Although for the aqueous environment the phase jump is slightly smoothened, it is still prominent (Fig. 2b). The total phase variation Δ is about 240 deg. and 270 deg. in the case of air and water environments, respectively. For our parameters of the system the phase jump was much sharper for air ambience compared to the aqueous one.

**Figure 3:**
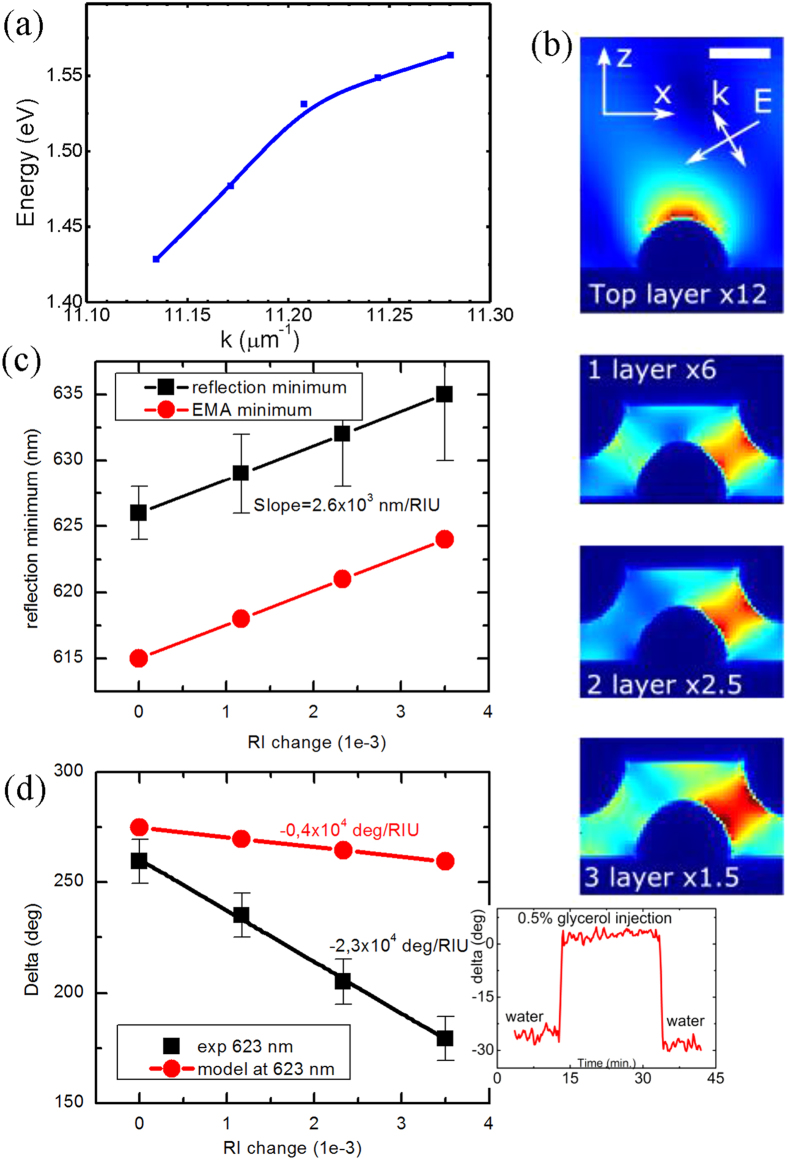
Properties of resonances in 3D Woodpile photonic crystal arrays, assessment of sensitivity in biosensing. (**a**) Dispersion curve constructed on the basis of experimental data (combinations of the resonance angle of incidence and wavelength). (**b**) Distribution of electric field inside the metamaterials structure obtained from FDTD simulations; Calibration curves for spectral (**c**) and phase (**d**) responses as a function of refractive index change caused by the addition of different glycerol concentrations. Experimental and theoretical data are presented by black and red lines, respectively. The inset shows a typical shift of phase Δ when 0.5% of glycerol is added to buffer water solution pumped through the metamaterial structure.

In order to assess the potential of the 3D woodpile-based plasmonic crystals in biosensing, it is important to clarify the nature of the plasmon modes responsible for the observed resonances. First of all, elongated metal-coated woodpiles are expected to support delocalized (propagating) plasmons, which can be excited e.g., due to the presence of periodic modulations, similar to the case of nanohole arrays in thin metal films^14^,^15^. The excitation of such delocalized plasmonic mode is indeed confirmed by a characteristic ascending course for optical energy dispersion *E(k*_*x*_), shown in Fig. 3a, which is obtained on the basis of experimentally determined conditions of resonance generation (combinations of the resonance angle of incidence and wavelength). It is interesting that the whole range of optical energy variations appears to happen under a very narrow range of wave numbers Δ*k* ~ 0.11 μ m^−1^, corresponding to a very narrow range of angles of incidence (Δ*θ* <1–2° deg.). Out of this narrow range of angles, the plasmons are not excited and the resonances disappear. On the other hand, the presence of nodes in woodpile contacts can favor the excitation of localized plasmons. In this case, the Finite-difference time-domain (FDTD) approach can be used to determine the localization of the electric field caused by the LPR excitation. Here, for relatively thin Ag coatings (<20 nm), metal-covered woodpile photonic crystals can be modelled as core-shells^26^, but due to the excessive number of interfaces involved, the FDTD consideration is typically limited to 2D structures. In our case, the thickness of the metal coating (30–40 nm) is larger than the skin depth for the electromagnetic field (~20 nm for Ag in the visible range). Therefore, to a first approximation we may treat the woodpiles as solid metal nanorods. Such an approach drastically simplifies FDTD simulations and makes possible the analysis of the electric distribution inside the 3D plasmon crystal matrix. We employed a FTDT package from Lumerical Solutions Inc., assuming that the angle of incidence of the p-polarized beam on the structure is 60°. The result of such a numerical description is given in Fig. 3b. One can see that the electric field is indeed concentrated at the lattice nodes, corresponding to the positions of the transverse bars with respect to the light incidence plane. Such a concentration of the electric field can only be explained by the generation of localized plasmon resonances. For the first layer, the electric field is concentrated at the peaks of the nodes, while for the inner layers, the main point of field concentration shifts from the top of the nodes, which is probably explained by the inclined geometry of the illumination of the woodpiles by external light. As it follows from our analysis, a remarkable concentration of electric field takes place only for the top 8–10 woodpile layers. However, one has to notice that the approximation of Ag-coated woodpiles as solid metal nanorods presents an extreme case, which is characterized by maximum light absorption. The extrapolation of the FDTD approach to real structures having a finite thickness of metal coating is supposed to increase the penetration depth of the light electric field towards much deeper layers. A rigorous consideration of structures having precise experimental parameters is now in progress and will be published elsewhere.

It is known that hybrid metal/dielectric systems can be fairly well described by employing the effective medium approximation (EMA)^27^,^28^,^29^. In fact, by using this approach one designs an effective medium with a complex index of refraction, which could provide a similar optical response to what is observed in the experiments^27^. In this case, the Maxwell-Garnett EMA approximation is typically applied to simulate isolated conductive elements^27^,^28^,^29^, while the Bruggeman EMA approximation appears to be more efficient when metal elements are electrically connected^30^. EMA is typically applied to structures with the size of features a ≪ λ , but surprisingly it is still valid for plasmonic systems with a ~ λ 27. Since the 3D woodpile photonic crystal structure used in this study presents a combination of electrically connected metal-covered nanopile (nanorod) elements, the application of the Bruggeman approximation looks more adequate. Indeed, our tests showed that this approximation could provide a fairly precise description of the optical properties of silver-coated woodpile metamaterial structures. In this case, the effective dielectric constant of the hybrid medium (silver-coated piles with air filled gaps) is approximated by the following Bruggeman equation:


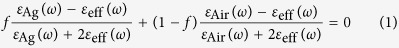


where *ε*_*Ag*_, *ε*_*Air*_*, ε*_*eff*_ *a*re the dielectric constants of Ag, air, and the hybrid effective medium, respectively; *f* is the fraction of silver in the net volume of the structure. Using the General Oscillator model fit for Ag optical constants from experimentally obtained data (a part of Woollam CompleteEase software), we could select parameters of the effective medium matching the experimental optical response, in terms of ellipsometric parameters Ψ and Δ. In our tests, this result was achieved by designing a hybrid Ag – dielectric medium with *f* =  0.1 (10% of Ag in air or water ambience; optical parameters were taken from Palik’s book^31^). As shown in Fig. 2(a,b), despite some minor deviations of the theoretical curves from the experimental ones out of resonance (especially for water), the Bruggeman EMA approximation demonstrates a very good accuracy in characterizing both the position and the line shape of the resonances, as well as in the description of related phase jumps.

In order to estimate the sensitivity of plasmonic modes excited in 3D woodpile structures, we carried out a series of model sensing tests. We used a well-established glycerine model, in which different concentrations of glycerine are added to water in order to provide controllable changes of RI^11^,^21^. Aqueous solutions of glycerine were pumped through a flow cell by a peristaltic pump and brought into contact with the metamaterial structure. The block containing the metamaterial slide and the flow cell was placed onto the ellipsometer platform and examined. Figure 3c shows the response of spectral position of the resonance to variations of RI, obtained from the experiment and theoretical modelling using the Bruggeman EMA model. It can be seen that an increase of refractive index (RI) due to the glycerine addition leads to a shift of the resonance. Here, under a relatively large range of RI variations, the structure demonstrated almost linear dependence of the resonant minimum position on RI change. The spectral sensitivity is estimated to reach 2600 nm/RIU, which is almost one order of magnitude higher than in cases of NHA and LPR counterparts. It is important that theoretical estimations by EMA model predict the same spectral sensitivity to RI variations (Fig. 3b), suggesting that the metallized woodpile arrays operates as an effective medium with an appropriate composition of metal (Au woodpiles) and dielectric (water-filled gaps between woodpiles) constituents. As follows from Fig. 3d, the phase also demonstrates a linear dependence to RI changes within this range. In particular, the injection of a very small concentration of glycerol (0.5%) corresponding to a change of RI by 10^−3^ RIU caused a shift of phase by more than 30° deg., which was far beyond the noise level in the system. Thus, the phase sensitivity was higher in our case than 3·10^4^ deg. of phase shift per RIU change, which is slightly lower that values obtained with DC- LPR in 2D plasmonic nanodot arrays (>2*10^5^ deg. of phase per RIU)^11^,^21^, but comparable or better than relevant values for SPR for gold films^19^ or hybrid metal film/graphene architectures^32^,^33^. Interestingly, modelling using the EMA approximation predicted a 3-fold lower sensitivity compared to the experiment. We believe that such a discrepancy is explained by imperfections in the EMA approach, as it does not take into account topological phase properties.

Thus, plasmon modes excited over 3D plasmon crystal metamaterials can break the diffraction-related limitation of 2D periodic structures and provide spectral sensitivity of 2 600 nm/RIU, which is comparable with best values for thin film-based SPR in the Kretschmann-Raether prism geometry and outperforms relevant sensitivities of LPRs and SPPs in alternative nanoscale architectures of nanoparticle (250–500 nm/RIU)^7^,^9^,^11^,^13^ and nanohole (300–400 nm/RIU)^14^,^15^ arrays, respectively. It is interesting that a certain increase of spectral sensitivity enhancement was earlier reported for NHA arrays under conditioning of their quasi-3D dimensionality^34^,^35^. In particular, the sensitivity could be increased up to 700–800 nm/RIU and 1100 nm/RIU under the addition of a second, physically separate level of isolated gold disks at the bottoms of the nanoholes^34^, or by using metal-coated substrate-supported quasi-3D-dimentional half-sphere arrays^35^, respectively.

We suppose that the photon crystal metamaterial structures, described in our work, present the next step of dimensional scaling toward true 3D nanoarchitectures, enabling the excitation of plasmon modes over the volume of the metamaterial matrix. We believe that with the help of metallized woodpiles we artificially create an effective medium composed of metal (Ag-coated woodpiles) and dielectric (gaps between the woodpiles) sub-media. The illumination of such structures by light causes the excitation of delocalized plasmon oscillations (electric currents) over electrically connected elements of the medium (metallized woodpile structures), which leads to a drastic loss of effective reflectivity at an appropriate combination of angle of light incidence and the pumping wavelength. Such plasmon mode provides a much improved response to refractive index variations, as the sensed dielectric environment becomes a part of the artificially formed effective metal-dielectric medium. The situation is in many respects similar to our previous article on plasmon nanorod metamaterials^6^. In this paper, by using a “forest” of densely packed long (~350–450 nm) gold nanorods, oriented perpendicularly to a glass substrate, we also created a quasi-3D effective medium with metal and dielectric constituents, although the thickness of this layer was much smaller (<450 nm). Plasmonic modes excited over such a “forest” also provided unexpectedly high response to RI variations (up to 30000 nm/RIU), which was in accordance with EMA-based estimations. In our plasmon crystal metamaterial case, we expect a further enhancement of sensitivity above 10 000 nm/RIU with a proper optimization of the 3D plasmonic crystal geometry. In particular, our estimations and preliminary tests show that such a gain of sensitivity can be achieved through the decrease of plasmon crystal unit cell and slight modifications of its architecture. It is important that in contrast to plasmonic nanorod arrays^6^ and other nanohole^14^,^15^ or nanoparticle^7^,^9^,^11^,^13^ structures plasmon modes excited in 3D photonic crystal structures can combine high spectral sensitivity and extremely promising characteristics of light phase conditioned by extreme darkness of the observed resonances. Indeed, the light darkness in resonances provided prominent phase singularities, yielding the sensitivity of more than 3*10^4^ Deg. of phase per RIU. As shown in ref. 21, this level of sensitivity is enough to enable label-free single molecule detection. Therefore, the proposed sensor nanoarchitecture matches earlier stated criteria for desired parameters of future sensor prototypes, namely the combination of ultrahigh point sensitivity (provided by the phase interrogation) and fairly high spectral sensitivity for a wider dynamic range of measurements (provided by the spectral interrogation).

In summary, we showed that the employment of 3D nanoarchitectures of plasmon excitation can break the diffraction-related limitation of 2D periodic structures and thus obtain a much improved spectral sensitivity (2600 nm/RIU), as well as a prominent phase response (3*10^4^ Deg. of phase per RIU). The sensitivity enhancement was demonstrated using Woodpile-based photonic crystal metamaterials, but we believe that similar effect can be obtained with alternative 3D architectures (nanorod etc) constituting effective metal/dielectric medium. One of the key advantages of the proposed metamaterial-based transducer geometry consists in the large area for biomolecule immobilization offered by the 3D matrix. This enables the implementation of new sensing geometries and strategies, not feasible with film-based SPR or 2D LPR. Indeed, by functionalizing the woodpile blocks and immobilizing a receptor on their surfaces, one can follow the binding of a selective analyte with the receptor inside the woodpile matrix. The considerably increased surface area given by the metamaterial topography significantly increases the amount of biomaterial that can be incorporated into the matrix within the available probe depth, maximizing the “biological” sensitivity of the system. Furthermore, the distance between the woodpile blocks can be selected to match the size of biological species of interest, giving access to a further size selectivity option that is important for many tasks in immunoassays and virus and protein detection. In general, relatively large openings in the woodpile array could contribute to a good transport of biological species, which is not always the case when porous nanomaterials are used as bioimmobilization templates. Finally, a strong field enhancement at some points of the metamaterial matrix makes possible the involvement of an additional Surface Enhanced Raman Scattering (SERS) channel, which can be used in parallel with the main optical transduction channel.

## Methods

### Materials

The materials used here are a zirconium-silicon organic-inorganic hybrid materials doped with tertiary amine moieties. Their synthesis has been described previously^23^. The main material components are methacryloxypropyl trimethoxysilane (MAPTMS), zirconium propoxide (ZPO, 70% in propanol) and 2-(dimethylamino) ethyl methacrylate. (DMAEMA) is added to act as a quencher, and to provide the metal-binding moieties. Michler’s ketone, 4,4-bis(diethylamino) benzophenone (BIS) is used as a photoinitiator. MAPTMS is first hydrolyzed using HCl solution (0.1 M) at a 1:0.1 ratio. After 5 minutes, the hydrolyzed MAPTMS is slowly added to the ZPO at 8:2 molar ratio. After stirring for 15 minutes, DMAEMA is added. The MAPTMS:DMAEMA molar ratio is 7:3. Finally, the photoinitiator, at a 1% w/w concentration is added to the mixture. After stirring for a further 20 minutes, the composite is filtered using a 0.22 μ m syringe filter.

The samples are prepared by drop-casting onto 100 micron-thick silanized glass substrates, and the resultant films are dried in an oven at 50 °C for 10 minutes before photopolymerization by DLW. After the completion of the component build process, the samples are developed for 20 minutes in a 30:70 solution of 1- propanol:isopropanol, and further rinsed with isopropanol.

All chemicals are obtained from Sigma-Aldrich and used without further purification.

### Fabrication of woodpile metamaterial structures

The structures fabricated are PhCs using the fcc woodpile geometry, with intralayer erodicity of 700 nm. The experimental setup employed for their fabrication has been described previously^23^,^24^. A Ti: Sapphire femtosecond laser (Femtolasers Fusion, 800 nm, 75MHz, <20 fs) is focused into the photopolymerisable composite using a high numerical aperture focusing microscope objective lens (100×, N.A. = 1.4, Zeiss, Plan Apochromat). Sample movement is achieved using piezoelectric and linear stages, for fine and step movement, respectively (Physik Instrumente). The average power used for the fabrication of the high-resolution structures is 4.0 mW, measured before the objective, while the average transmission is 20%. The scanning speed is always set to 20 μ m/s.

The PhCs used for these experiments consisted of 7 unit cells, and average rod thickness (including the silver layer) was about 280 nm.

### Metallization of woodpile metamaterial arrays

The metallization process followed here is a modification of the one described in ref. 23. It consists of three steps: seeding, reduction and silver plating.

#### Seeding

The samples were immersed in a 0.05 mol/L AgNO_3_ aqueous solution at room temperature for 38 hours minimum. This was followed by thorough rinsing with double distilled (d.d.) water, by immersing the sample twice in a water container, with fresh water for each immersion. They were left to dry at room temperature.

#### Reduction

An aqueous sodium borohydride (NaBH_4_) solution 6.6 M was prepared some hours (>4 h) before the immersion of the samples. The solution was very well mixed and kept uncovered for a couple of hours to get rid of trapped air bubbles. The samples were subsequently dipped in the solution for 22 hours to reduce the silver ions and form silver nanoparticles. The samples were subsequently washed thoroughly in fresh d.d. water and left to dry.

#### Silver plating

A 0.2 M AgNO_3_ aqueous solution was mixed with 5.6% NH_3_ (28% in water) and 1.9 M glucose (C_6_H_12_O_6_ >  98%) as a reducing agent, at volumetric ratio 5:3:8.

The samples were immersed in the solution for a few minutes, and were removed before it became dark. In the meanwhile, a fresh solution was prepared to replace the old one. This process was repeated two times. The samples were subsequently washed twice by immersion in fresh d.d. water for a few minutes.

### Optical characterization

The samples were characterized in a Woollam M-2000 ellipsometer, which monitors phase-polarization properties of light reflected from a thin film in order to determine its optical parameters. Parameters of light reflected from a film can be derived through normalized electric field components *r*_*p*_ and *r*_*s*_ (complex quantities), which correspond to the field components parallel and perpendicular to the plane of light incidence. The ellipsometry measures the complex reflectance ratio ρ , which is the ratio of *r*_*p*_ over *r*_*s*_, and can be decomposed into amplitude and phase components:


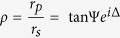


where the real part tan(Ψ ) is the amplitude ratio upon reflection, and the imaginary part Δ is the phase shift (difference between the phase of the *r*_*p*_ and *r*_*s*_ components). Measured parameters can then be routinely converted to more the conventional *r*_*p*_ and *r*_*s*_.

## Additional Information

**How to cite this article**: Aristov, A. I. *et al.* 3D plasmonic crystal metamaterials for ultra-sensitive biosensing. *Sci. Rep.* **6**, 25380; doi: 10.1038/srep25380 (2016).
